# Phase-locking of epileptic spikes to ongoing delta oscillations in non-convulsive status epilepticus

**DOI:** 10.3389/fnsys.2013.00111

**Published:** 2013-12-16

**Authors:** Rikkert Hindriks, Hil G. E. Meijer, Stephan A. van Gils, Michel J. A. M. van Putten

**Affiliations:** ^1^Department of Clinical Neurophysiology, MIRA-Institute for Biomedical Technology and Technical Medicine, University of TwenteEnschede, Netherlands; ^2^Computational Neuroscience Group, Department of Information and Communication Technologies, Center for Brain and Cognition, Universitat Pompeu FabraBarcelona, Spain; ^3^Department of Electrical Engineering, Mathematics and Computer Science, MIRA-Institute for Biomedical Technology and Technical Medicine, University of TwenteEnschede, Netherlands; ^4^Department of Neurology and Clinical Neurophysiology, Medisch Spectrum TwenteEnschede, Netherlands

**Keywords:** absence status, delta oscillation, absence seizure, spike-wave discharge, phase-locking, thalamo-cortical system

## Abstract

The EEG of patients in non-convulsive status epilepticus (NCSE) often displays delta oscillations or generalized spike-wave discharges. In some patients, these delta oscillations coexist with intermittent epileptic spikes. In this study we verify the prediction of a computational model of the thalamo-cortical system that these spikes are phase-locked to the delta oscillations. We subsequently describe the physiological mechanism underlying this observation as suggested by the model. It is suggested that the spikes reflect inhibitory stochastic fluctuations in the input to thalamo-cortical relay neurons and phase-locking is a consequence of differential excitability of relay neurons over the delta cycle. Further analysis shows that the observed phase-locking can be regarded as a stochastic precursor of generalized spike-wave discharges. This study thus provides an explanation of intermittent spikes during delta oscillations in NCSE and might be generalized to other encephathologies in which delta activity can be observed.

## Introduction

Non-convulsive status epilepticus (NCSE), also known as petit mal status, absence status, or ictal confusion, refers to a prolonged state in which the subject's EEG displays epileptiform activity (Brenner, 2004; Kaplan, 2006). It frequently occurs in elderly patients both with and without a history of epileptic seizures (Ellis, 1978; Lee, 1985; Bauer et al., 2007). NCSE is a highly heterogeneous pathological condition whose clinical manifestations include confusion, euphoria, attentional and memory problems, unconsciousness (van Putten and van Putten, 2007), visual and auditory hallucinations, and paranoia (Granner, 1994). In fact, in the past, patients in NCSE have mistakingly been diagnosed as suffering from psychiatric syndromes, in particular psychosis, and manic-depression (Ellis, 1978; Brenner, 2004). The etiology of NCSE ranges from structural CNS lesions and toxic-metabolic encephalopathies, to CNS infections, hypoxic-ischemic injury, and anti-epileptic drug withdrawal (Treiman and Walton, 1990; Tay et al., 2006).

Although NCSE is a highly dynamic process, as reflected in the progression of EEG patterns (Treiman and Walton, 1990) four main pattern types have been identified: typical, atypical, multiple spike-and-wave discharges, diffuse rhythmic delta activity (DRDA) with intermittent spikes (Ellis, 1978; Granner, 1994; Brenner, 2004; Tay et al., 2006). DRDA, however, can also be observed without spikes (Uthman and Bearden, 2008) suggesting a possible relation with other forms of pathological delta activity, for example, diffuse delta slowing and intermittent rhyhmic delta activity, which can be observed in many types of encephalopathies (Smith, 2005; Kaplan and Birbeck, 2006; Brigo, 2011). Most studies, however, are empirical and not much is known about the underlying mechanisms and the structures involved (Brigo, 2011).

In contrast to SWDs observed during generalized absence seizures, which are thoroughly studied using macroscopic models (Robinson et al., 2002; Suffczynski et al., 2004; Breakspear et al., 2006; Rodrigues et al., 2006, 2009; Roberts and Robinson, 2008; Kim et al., 2009; Marten et al., 2009) we are not aware of any theoretical studies focusing on DRDA (with or without intermittent spikes). Some modeling is done, however, on frontal intermittent rhythmic delta activity, which may be relevant for DRDA during NCSE as well (Stam and Pritchard, 1999). In Stam and Pritchard (1999) the authors used two coupled neural masses to show that frontal intermittent rhythmic delta activity possesses limit-cycle as opposed to fixed-point dynamics. Nevertheless, their modeling suggests, or presupposes, the existence of local delta generators, whose synapto-dendritic time-constants determine their oscillation frequency. In this study we take a different approach and argue that DRDA is tightly linked to SWDs. Since more is known on the mechanisms underlying the generation of SWDs, in particular their connection to pathological spindle activity (Kostopoulos et al., 1981; Wang et al., 1991; Steriade et al., 1993; von Krosigk et al., 1993; Bal and von Krosigk, 1995; McCormick and Bal, 1997; Robinson et al., 2001) this might elucidate some aspects of DRDA. We thus concentrate on the role of the thalamus, which has already been shown to be involved in NCSE (Druga et al., 2001; Fabene et al., 2003; Hamani et al., 2008).

Using an EEG recording of a patient in NCSE, we show that the spikes are phase-locked to the DRDA. Moreover, we employ an established model of thalamo-cortical dynamics (Robinson et al., 2001; Rennie et al., 2002) to suggest physiological mechanisms underlying this phenomenon. It is argued that the spikes reflect negative stochastic input to thalamo-cortical relay neurons which can be transmitted only during a narrow temporal window of the delta cycle. This window leads to the appearance of phase-locked spikes in the EEG. We further argue that the phase-locked spikes can be regarded as a stochastic precursor generalized SWDs. The findings reported in this study link generalized absence and tonic-clonic seizures (Breakspear et al., 2006) to NCSE and thereby show that the employed model might provide a common theoretical framework for studying different epileptic syndromes.

## Materials and methods

### Thalamo-cortical model of the EEG

In this study we use the thalamo-cortical meanfield model of EEG generation developed in (Robinson et al., 2001, 2002; Rennie et al., 2002). The model comprises four types of neural populations, consisting of cortical pyramidal, cortical inhibitory, thalamo-cortical relay, and thalamic reticular neurons, which are denoted by the subscripts *e, i, s*, and *r*, respectively. The model describes the dynamics of the average membrane potentials *V*_*k*_ and firing rates *Q*_*k*_ of the different populations *k* = *e, i, s, r*. The firing rates depend on the mean membrane potentials through the activation function

(1) $$S(V_{k})=\frac{Q_{\max}}{1+e^{-(V_{k}-\theta )/\sigma }},$$

where *Q*_max_ is the maximal average firing rate, θ is the average spike threshold, and σ quantifies the variation of spike thresholds of population *k*.

**Figure 1:**
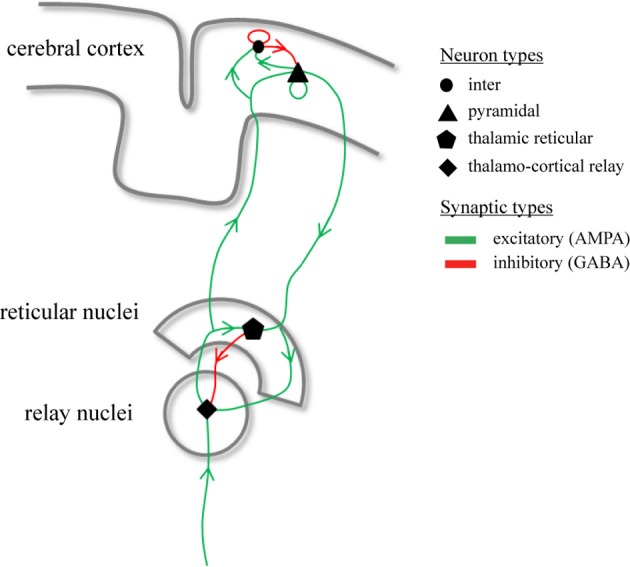
**Synaptic organization of the thalamo-cortical model**. Illustrated are the synaptic pathways that connect the different neuronal populations within the model. The model contains four types of neuronal populations which are connected through excitatory (green) and inhibitory (red) synaptic projections.

Mean firing rates arriving at the neural populations induce a synapto-dendritic impulse response *h* given by

(2) $$h(t)=\frac{\alpha \beta }{\beta -\alpha }\left(e^{-\alpha t}-e^{-\beta t}\right),$$

where β > α and β and α denote the synapto-dendritic rise and decay rates, respectively (Robinson et al., 1997). The mean firing rate *Q*_*e*_ of cortical pyramidal neurons spreads over the cortical sheet via long-range cortico-cortical fibers according to

(3) $$\frac{d^{2}}{dt^{2}}\phi_{e}+2\gamma \frac{d}{dt}\phi_{e}+\gamma^{2}\phi_{e}=\gamma^{2}Q_{e},$$

where ϕ_*e*_ (*t*) is the mean firing rate of cortical pyramidal neurons propagated to distant pyramidal neurons, γ = *v*/*l* is the cortical damping rate, *v* the axonal propagation velocity, and *l* the characteristic axonal length of cortical pyramidal neurons (Robinson et al., 1997). The absence of a spatial derivative in (3) implies that we restrict to spatially-homogeneous dynamics on the cortical sheet. Since we focus on modeling DRDA and generalized SWDs which, by definition, are global cortical phenomena, this restriction is justified (Robinson et al., 2002; Breakspear et al., 2006; Hindriks and van Putten, 2012). The strength of synaptic connections from neurons of type *l* to neurons of type *k* is denoted by ν_*kl*_ (Robinson et al., 1997). Figure 1 provides an illustration of the synaptic organization of the model. Following (Robinson et al., 2002) we assume that experimentally recorded EEG signals are proportional to −ϕ_*e*_(*t*). Table 1 lists the parameter values for which the model displays delta oscillations and we will identify this parameter regime with DRDA during NCSE.

**Table 1:** **Model parameters, their symbols, and nominal values**.

**Parameter**	**Symbol**	**Nominal value**
Maximal firing rate	*Q*_max_	250 s^−1^
Average spike-threshold	θ	15 mV
Spike-threshold deviation	σ	3.3 mV
Synaptic decay rate	α	50 s^−1^
Synaptic rise rate	β	200 s^−1^
Synaptic strength from e to e neurons	ν_*ee*_	1 mV s
Synaptic strength from i to e neurons	ν_*ei*_	−1.8 mV s
Synaptic strength from s to e neurons	ν_*es*_	3.2 mV s
Synaptic strength from i to i neurons	ν_*ii*_	−1.8 mV s
Synaptic strength from e to i neurons	ν_*ie*_	1 mV s
Synaptic strength from s to i neurons	ν_*is*_	3.2 mV s
Synaptic strength from r to s neurons	ν_*sr*_	−0.8 mV s
Synaptic strength from e to s neurons	ν_*se*_	2.2 mV s
Synaptic strength from s to r neurons	ν_*rs*_	0.6 mV s
Synaptic strength from e to r neurons	ν_*re*_	1.6 mV s
Average noise level	ν_*sn*_ϕ_*n*_	2.0 mV
Noise standard deviation	σ_*n*_	0.2 mV
Cortico-thalamic delay	τ	0.04 s
Cortical damping rate	γ	100 s^−1^

### Clinical EEG data

On April 1, 2011, a 65-year old patient was seen at the emergency department of the hospital Medisch Spectrum Twente, Enschede, The Netherlands. History taking was hardly possible, due to a severe dyspnoea. Oxygen saturation was 88%, with an arterial *pO*_2_ = 7.3 kPa, *pCO*_2_ = 6.8 kPa and a *pH* = 7.50. He was initially diagnosed with a decompensatio cordis and treated with diuretics. His condition hardly improved, however, and he remained confused. Soon after, he developed a pneumonia with a respiratory insufficiency, for which he was transferred to the Intensive Care Unit on April 3, 2011. He was intubated and mechanical ventilation was started. He was sedated with continuous infusion of propofol. After a few days, his respiratory condition gradually improved, and sedation was stopped. A few hours later, he suffered from a generalized tonic-clonic seizure, for which the neurologist was consulted. On clinical examination, his Glasgow coma score was minimal. There were no abnormal eye movements, and pupil size was normal, with intact reactions to light. A brain CT showed moderate generalized atrophia and diffuse white matter abnormalities, without any signs of recent ischaemia or hemorrhage. Under suspicion of a possible encephalitis, a lumbar puncture was performed. The opening pressure was normal, and the cerebrospinal fluid revealed no significant abnormalities.

He was treated with diphantoine, but consciousness did not return. The differential diagnosis included a non-convulsive status epilepticus (NCSE) and continuous EEG recording was started. This showed rhythmic, high voltage (150 μ V) delta activity, with a left hemispheric dominance. Sometimes, spikes were observed, as well. This pattern was interpreted as electroencephalopgraphic seizure activity. After about 40 min, the rhythmic delta activity evolved into rhythmic spikes, and the patient suffered from a second generalized seizure. Propofol was restarted, but non-convulsive seizure activity continued. Therefore, midazolam and sodium valproate were added, too. Eventually, after 2 days, all epileptiform discharges disappeared.

After gradual reduction of the sedation with propofol and midazolam, our patient eventually recovered consciousness. Initially, he showed a severe bradyphrenia, with a mild right-sided hemiparesis. A repeat CT cerebrum showed two subcortical infarctions in the right hemiphere, that did not explain his mild right-sided paresis. For more than a week, he was successfully weaned from the ventilator and his condition further improved. He was discharged from our ICU on April 14, 2012. In sum, this 65-year old patient suffered from both convulsive and non-convulsive seizure activity, where the EEG showed rhythmic delta activity with intermittent spikes. This is a relatively rare EEG pattern, that should not be interpreted as post-ictal slowing, but as an ictal phenomenon. All data were obtained as part of our routine patient care.

**Figure 2:**
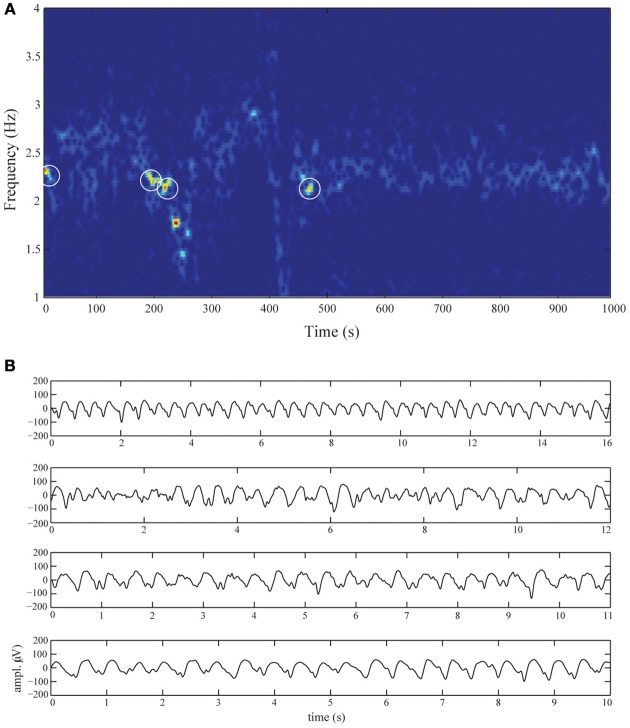
**Diffuse rhythmic delta activity during non-convulsive status epilepticus. (A)** Spectrogram of electrode Fz over the entire recording-length. The activity is almost completely confined to frequencies in the delta band (1–3 Hz). The four white circles schematically denote the selected epochs. These epochs are shown in **(B)**, displaying DRDA with frequencies between 2 and 2.5 Hz and amplitudes up to 150 μ V. The oscillations are dispersed with intermittent spikes.

The EEG was recorded using 21 silver-silverchloride cup electrodes placed on the scalp according to the international 10/20 system and using the average montage. Recordings were made using a Neurocenter EEG recording system (Clinical Science Systems, Voorschoten, The Netherlands). Electrode impedances were below 5 kΩ and the sampling frequency was set to 250 Hz. We selected a 1000-s time-series of electrode Fz displaying DRDA activity. The corresponding spectrogram is shown in Figure 2A. The time-series were filtered between 0.5 and 20 Hz using a fourth-order zero-phase Butterworth filter. For subsequent analysis, we selected the high signal-to-noise epochs marked by white-circles. The length of the selected epochs where 16, 12, 11, and 10 s. The epochs are shown in Figure 2B.

### Phase-locking statistics

To quantify the extent of locking of spikes to ongoing delta oscillations, both in real EEG time-series as well as simulated time-series, we proceed as follows. Let *x* = *x*_1_, …, *x*_*n*_ denote the samples of a recorded or simulated time-series. We extract the spike locations by first selecting all local maxima and then discarding those above a chosen threshold. The threshold effectively isolates the spikes from the peaks of the delta oscillations. Let *t*_1_, …, *t*_*k*_ be the samples that correspond to the spikes. To identify these samples with phases of the ongoing delta oscillations, we first bandpass-filter *x* within the frequency band 1–3 Hz using a second order zero-phase Butterworth filter, thereby obtaining a filtered time-series *y* = *y*_1_, …, *y*_*N*_. The instantaneous phases ϕ_1_, …, ϕ_*N*_ of *y* are obtained through the analytic signal *y*^*A*^ of *y*, which is defined as

(4) $$y^{A}=y+iy^{H},$$

where *y*^*H*^ denotes the Hilbert transformation of *y* (Pereda et al., 2005) and then taking the radial angles

(5) $$\phi_{n}=\text{arg}(y_{n}^{A}),$$

for *n* = 1, …, *N* in the interval [−π, π]. The phases of the spikes ψ_*m*_ are now given by ψ_*m*_ = ϕ_*t*_*m*__, for *m* = 1, …, *k*.

To quantify the extent of phase-locking, we compute the variable *z*, which is defined as

(6) $$z=\frac{1}{k}\sum_{m=1}^{k}e^{i\psi_{m}},$$

and takes on values in the unit-disk in the complex plane. The extent of phase-locking is quantified by the *phase-coherence* Ω which is defined as

(7) Ω=|z|,

where the vertical bars denote absolute value. The phase-coherence Ω takes values in the interval [0,1], where Ω = 0 reflects absence of phase-locking and Ω = 1 reflects complete phase-locking. Furthermore, the *phase-angle* Ψ is defined as

**Figure 3:**
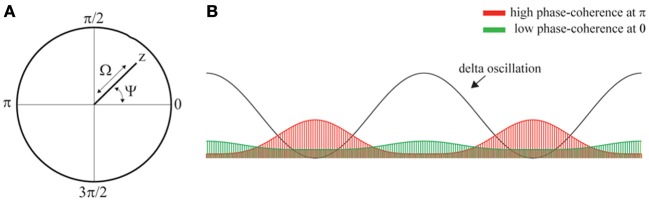
**Phase-locking of spikes to ongoing delta oscillations. (A)** Illustration of the variable *z*, phase-coherence Ω, and phase-angle Ψ which are obtained from *z* by taking its absolute value and argument, respectively. **(B)** llustration showing a delta oscillation and two phase-distributions (red and green areas). The red and green distribution results from phase-locking at phase π and 0, respectively. Thus, the location of the peak of the distributions determines the phase-angle Ψ. The heigth of the distributions determined the coherence, which is strong and weak the green and red distributions, respectively.

(8) Ψ=arg(z),

which takes values in the interval [0,2π] and specifies the phase of the ongoing delta oscillations at which the spikes concentrate. Figures 3A,B provide illustrations.

Since the distribution of phase-coherence values under the null-hypothesis of no phase-locking is unknown, we assess statistical significance through the use of appropriate surrogate data (Schreiber and Schmitz, 2000). The general idea behind surrogate data-testing is to repeatably construct time-series under the null-hypothesis, which are used to simulate the distribution of phase-coherence values under the null-hypothesis. These surrogate time-series should have the same statistical properties as the original time-series, but lack the property that is tested for, in this case phase-coherence between intermittent spikes and ongoing oscillations. Phase-coherence is regarded as significant with *p* = 0.05 if the phase-coherence of the original time-series is contained in the 95% upper-quantile of the simulated distribution of phase-coherence values. In the present context, appropriate surrogate time-series contain the same number and timing of intermittent spikes but the phase-structure of the ongoing delta oscillations is destroyed. This is done by randomizing the Fourier-phases of *x* while leaving the spike-times *t*_1_, …, *t*_*k*_ unchanged (Pereda et al., 2005). The surrogate distributions were based on 1000 randomizations of the recorded EEG time-series.

## Results

### Diffuse rhythmic delta activity

**Figure 4:**
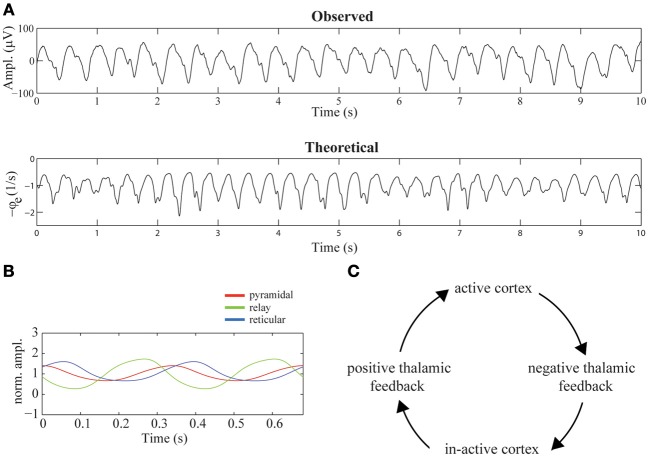
**Mechanism suggested to underly diffuse rhythmic delta activity. (A)** 10-s epochs of recorded (top-row) and simulated (bottom-row) EEG time-series. The simulated time-series were generated using the parameter values listed in Table 1. **(B)** The firing rates of cortical pyramidal neurons (red), thalamo-cortical relay neurons (green), and thalamic reticular neurons (blue) during two delta cycles. The firing rates are normalized by their mean values. **(C)** Illustration of the mechanism underlying the generation of delta oscillations in the model. The cycle of events corresponds to one delta oscillation and illustrates that it is generated by a periodic switching of the functional state of the thalamus, driven by periodic cortico-thalamic excitation.

The top-row of Figure 4A shows a 10-s sample of the first of the selected epochs. The time-series is dominated by delta oscillations with a frequency of about 2.5 Hz. Using the parameter values listed in Table 1, we simulated the time-series shown in the bottom-row of Figure 4A. Note the presence of strong delta oscillations similar to those observed in the recorded EEG time-series. In the recordings as well as in the simulated time-series, small epileptic spikes can be observed, occurring mostly on the down slopes of the delta oscillations. We treat these spikes in detail in Phase-locking of epileptic spikes and Physiological mechanism.

Figure 4B displays the average firing rates of pyramidal, thalamo-cortical relay, and thalamic reticular neurons during two delta oscillations, which, for better visibility, are normalized by their respective means. The mechanism underlying their generation is described in Robinson et al. (2001). In short, the mechanism consists of a periodic switching of the functional state of the thalamus, driven by periodic cortical feedback. Specifically, the thalamus periodically provides positive and negative feedback to the cortex, where *positive thalamic feedback* is characterized by high firing rates of relay neurons and near silence of reticular neurons and *negative thalamic feedback* is characterized by high firing rates of reticular neurons and near silence of relay neurons. A delta oscillation is generated as follows: active pyramidal neurons excite, after a delay τ, both reticular and relay neurons, which enhances their firing rates. The activation of reticular neurons inhibits the relay neurons, shifting the thalamus to its negative feedback state. The near silence of relay neurons, on their turn, leads, after a delay τ, to in-activation of pyramidal neurons. Again after a delay τ, the induced absence of cortical feedback to thalamus in-activates the reticular neurons, allowing the relay neurons to repolarize and become re-activated by the constant afferent excitation. The thalamus has now switched to its positive feedback state. After a delay τ, as a consequence of the activation of thalamic relay neurons, the cortex becomes active again, completing the delta cycle. This chain of events is illustrated in Figure 4C.

### Phase-locking of epileptic spikes

**Figure 5:**
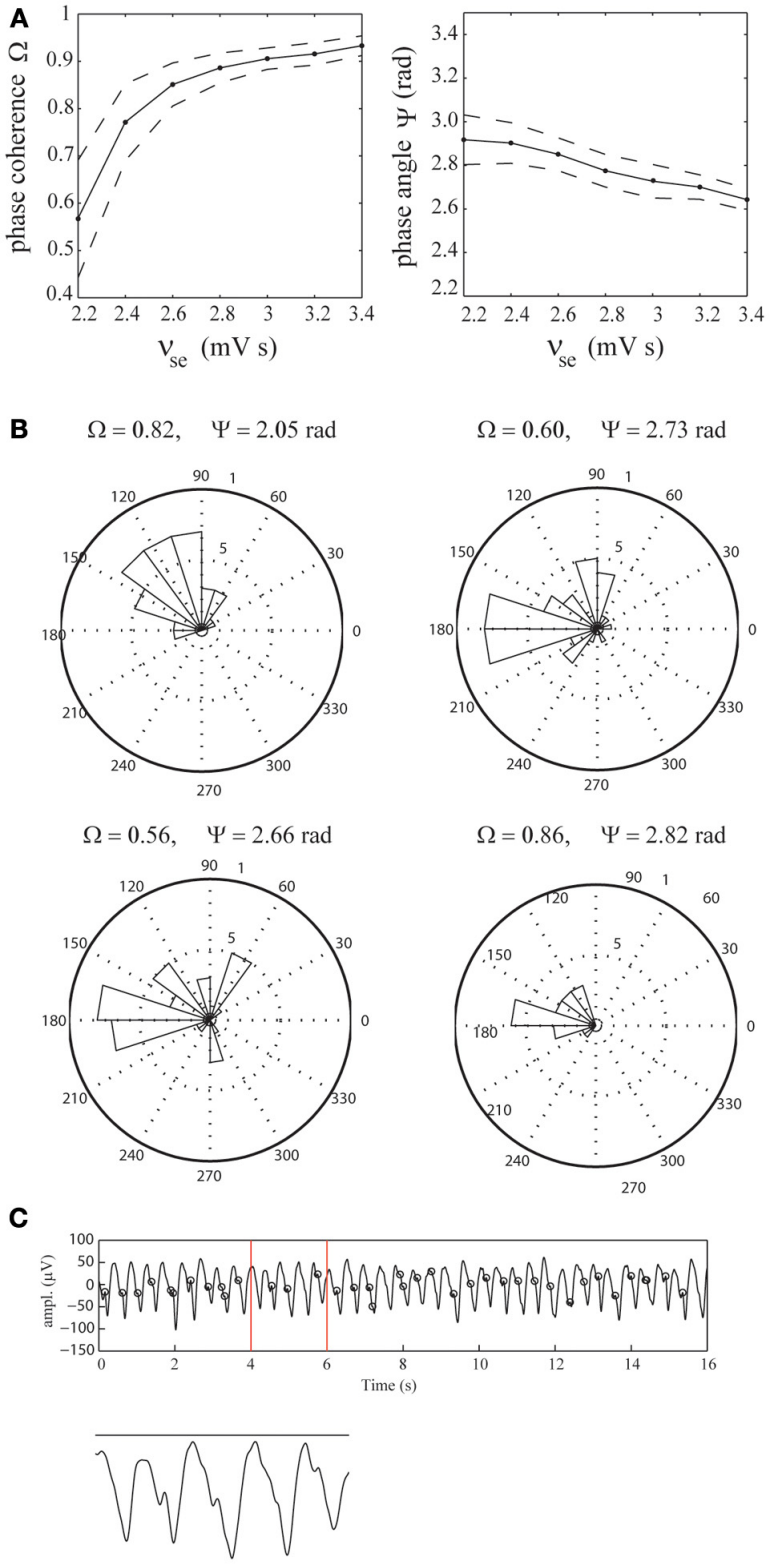
**Phase-locking of epileptic spikes. (A)** Mean phase-coherence Ω and phase-angle Ψ as a function of ν_*se*_ (solid lines), respectively. The parameter ν_*se*_ was increased from 2.2 to 3.4 mV s in steps of 0.2 mV s. For each value of ν_*se*_, the displayed values were obtained by computing the average phase-coherence and phase-angle over 100 simulations, each of length 18 s. Their standard deviations are indicated by dashed lines. **(B)** Radial distributions of the spike-phases for the four selected EEG epochs. **(C)** Illustration using the first epoch. Shown is the EEG time-series and the epileptic spikes (black circles). The vertical red lines mark the time-span of the close-up.

To assess the extent of phase-locking of the epileptic spikes observed in the simulated EEG time-series, we computed the mean phase-coherence Ω and phase-angle Ψ as a function of the efficacy ν_*se*_. For each value of ν_*se*_, the phase-coherence and phase-angle were computed as averages over 100 simulated time-series, each of 18 s duration. The results are displayed in Figure 5A, which shows that the phase-coherence increases as a function of ν_*se*_, reflecting increased locking of the spikes to the phase of the delta oscillations. Furthermore, it shows that the spikes phase-lock to the down slopes of the delta oscillations, just before their troughs and that the phase-angle slightly decreases for increasing values of ν_*se*_. Importantly, the dispersion of phase-coherence and phase-angle values around their respective means reflects stochasticity of the underlying phase-locking mechanism. Specifically, the spikes are not present in each delta oscillation, and if they are, they are dispersed around it (which is evident from the fact that the phase-coherence values are less than 1). The stochastic nature of the epileptic spikes is confirmed by the fact that in the absence of afferent noise (σ_*n*_ = 0), the spikes are absent (see Figure 7A).

To assess whether the epileptic spikes observed in the recorded EEG data are indeed phase-locked to the delta oscillations, we identified the spikes using a threshold of 30 μ V and estimated the phase-coherence and phase-angle from the four selected EEG epochs (see Figure 2B). We found that in all four cases, the phase-locking was significant (*p* < 0.001, *p* < 0.003, *p* < 0.001, and *p* < 0.004, respectively). The mean phase-coherence was 0.71 and the mean phase-angle 2.56 radians. Furthermore, all four phase-angles were contained in the interval [π/2,π], which corresponds to the lower part of the downslope of the delta oscillations. This agrees reasonably with the model simulations. Figure 5B shows the corresponding distributions of the spike-phases, relative to the phase of the delta oscillations for all four epochs. Figure 5C shows the first EEG epoch, together with the epileptic spikes (designated by black circles). Observe that, similar to the simulated time-series, the spikes are not present at every delta oscillation and are dispersed, indicating the stochastic nature of the underlying spike-generation mechanism.

### Physiological mechanism

The simulations described in Diffuse rhythmic delta activity show that there may be epileptic spikes at the downslopes of the delta oscillations (Figure 4A, bottom row). Here we describe the underlying physiological mechanism suggested by the model. In Phase-locking of epileptic spikes we have seen that the distribution of phase-angles of the spikes, although concentrated around a certain phase angle, has non-zero width, which reflects the stochastic nature of the spikes. Indeed, when the noise-level is set to zero, i.e., σ_*n*_ = 0, the spikes are absent (Figure 4B). This implies that they result from filtering of the stochastic afferent sensory input by the thalamo-cortical system and are not intrinsically generated by the model in this parameter regime.

**Figure 6:**
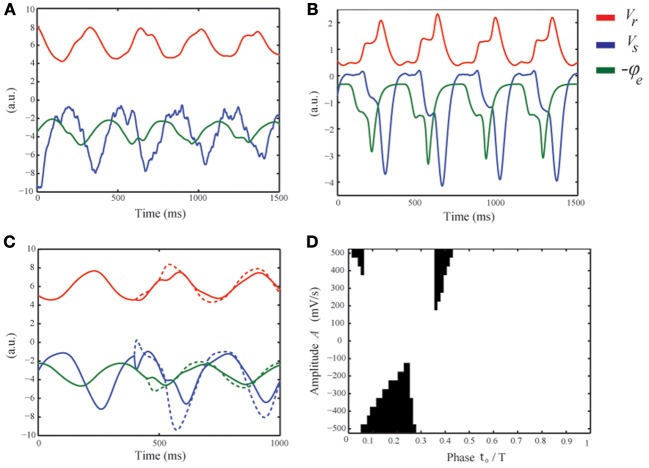
**Physiological mechanism suggested to underly the generation of intermittent epileptic spikes. (A)** Numerical simulation of the model equations with ν_*se*_ = 2.2 mV s, showing four delta cycles. (*V*_*s*_ blue, *V*_*r*_ red, and −ϕ_*e*_ green). **(B)** Similar to **(A)** but for ν_*se*_ = 4.4 mV s. For visibility, all variables have been scaled by the absolute value of their means. **(C)** For ν_*se*_ = 2.2 mV s and in the absence of noise, a perturbation with amplitude *A* = −500 (solid) and *A* = −500 (dashed) is applied. The negative perturbation leads to a spike, while the positive perturbation merely advances the delta cycle. **(D)** The black regions correspond to the values of *t*_0_ and *A* for which the applied perturbation leads to a spike.

First, we discuss the difference and similarity between these stochastic spikes and the deterministic spikes described in Robinson et al. (2001); Rennie et al. (2002); Breakspear et al. (2006). Figure 6A shows a simulation for ν_*se*_ = 2.2 mV s displaying DRDA with intermittent spikes. We first note that when the relay cells are active, i.e., *V*_*s*_ ≈ −1 mV, this is not sufficient to directly activate the reticular cells. Instead the pyramidal cells are driven first and subsequently the reticular cells are activated through cortical feedback. We observe an epileptic spike on two of the delta cycles shown. Observe that the spikes are preceded by a trough at the maxima of *V*_*s*_ i.e., during the active state of the thalamus. When ν_*se*_ is increased, the thalamic relay cells are slightly more active, sufficient to already interact with the reticular cells to generate a small plateau oscillation (see Figure 6B around *t* = 450 ms). In both cases the waveform of *V*_*s*_ displays a dip around its maximum. This negative deflection is transmitted to cortex and integrated by the pyramidal cells. If sufficiently large, this leads to a spike in −ϕ_*e*_, that is, in the EEG. For high values of ν_*se*_ there is little variation in the timing of the spike as it is generated in a deterministic fashion, i.e., by the interaction of relay and reticular cells. For lower values, the reticular cells are not sufficiently active and the stochastic fluctuations are responsible for the spikes. We conclude that the deflection in the waveform of *V*_*s*_ in the active state of the thalamus—either by fluctuations or by interaction with reticular cells—is the origin of the spike.

From these simulations we deduce three conditions for spikes to appear in the EEG. First, the relay cells should be in the active phase. Otherwise any fluctuation in *V*_*s*_ is ineffective as it is too negative so that their mean firing rate *Q*_*s*_ = *S*(*V*_*s*_) does not fluctuate. So we see that *dQ*_*s*_/*dV*_*s*_ i.e., the excitability of the relay cells, should be high. This is satisfied only during the active state of the thalamus. Second, at the beginning of the active phase, the mean afferent input dominates the drive of the relay cells, while at the end, the reticular cells exert their inhibition. In between, when *dQ*_*s*_/*dt* is small, fluctuations can appear in their firing rate *Q*_*s*_. Third, and similar to the first condition, the pyramidal cells should be sufficiently active. Indeed, also fluctuations in the waveform of the pyramidal cells should be visible in their firing rate *Q*_*e*_. The second condition informs us about the timing of the spikes. In the linear approximation, the time constants of the pyramidal cells and the cortical field are 1/α + 1/β and 2/γ, respectively. Thus, taking into account the thalamo-cortical delay τ, fluctuations in *V*_*s*_ in the active state of the thalamus appear about τ + 1/α + 1/β + 2/γ ≈ 85 ms later in the EEG. This argument is essentially the same as given in (Robinson et al., 2002) for estimating the period of petit-mal cycles. This limits the appearance of spikes to the second quarter of the delta cycle, i.e., in particular on the downslopes of the delta oscillations, in reasonable agreement with the experimental findings (see Figure 5B).

To understand the timing and nature of the spikes in more detail, we consider a deterministic perturbation impinging on relay cells of the form ϕ(*t*) = *v*_*sn*_ ϕ_*n*_ + *A*δ(*t* − *t*_0_), where *t*_0_ ranges from 0 to the period *T* of the delta oscillation. If *A* is sufficiently large and *t*_0_ is in the right temporal window, −ϕ_*e*_, that is, the EEG, exhibits a positive deflection, i.e., a spike (see Figure 6C). Choosing *t*_0_ in the beginning of the positive feedback phase of the thalamus, negative *A* merely delays the increase in ϕ_*e*_ as the potential *V*_*s*_ drops the relay cells exert almost no influence on the pyramidal neurons. Here, sufficiently large positive *A* leads to an initially faster increase in ϕ_*e*_, followed by a decrease as the delta oscillation lags behind, resulting in a spike. In the middle of the positive feedback phase, positive *A* advances the increase of ϕ_*e*_ and does not lead to spikes, while negative *A* slows down its increase as the input is temporally weaker and if *A* is sufficiently large, leads to a spike. Finally, choosing *t*_0_ near the end of the positive state of the thalamus, negative *A* reduces the already decreasing input even faster so that no spikes occur. Positive *A* can induce a spike if strong enough to increase ϕ_*e*_. Note that the first and third parts of the active thalamic state have a much smaller duration compared to the middle part. Figure 6D shows which values of *t*_0_ and *A* result in a spike (the black regions). For large positive *A* this is at the beginning and end of the active phase, but their temporal window is narrow. For negative *A*, the temporal window is wider and smaller absolute amplitudes lead to spikes. These regions are consistent with the above description and show that negative perturbations are most likey to result in spikes that are observable in the EEG.

### Relation to spike-wave discharges

**Figure 7:**
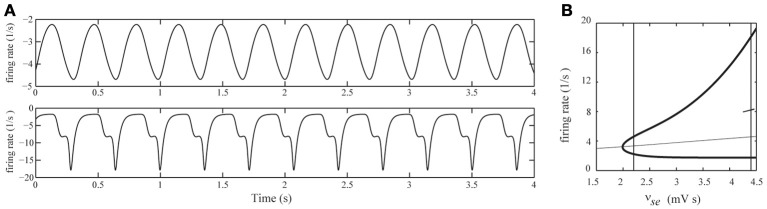
**(A)** Simulated EEG time-series for two different values of cortico-thalamic excitation (ν_*se*_). Top row: ν_*se*_ = 2.2 mV s, for which the EEG displays ongoing delta oscillations. Bottom row: ν_*se*_ = 4.4 mV s, for which the EEG displays 3-Hz spike-wave-discharges. In both simulations the afferent noise-level was set to zero (σ_*n*_ = 0). **(B)** Bifurcation diagram of the deterministic model for varying ν_*se*_. Steady state values of ϕ_*e*_ are indicated by the thin curve. Minimal and maximal values of ϕ_*e*_ of periodic solutions are indicated by the thick curves. The steady state is stable up to ν_*se*_ ≈ 2 mV s where a Hopf bifurcation occurs and a stable periodic solution appears. At ν_*se*_ ≈ 4.3 mV s the waveform shows an inflection point (see main text for details). The vertical lines correspond to the values ν_*se*_ = 2.2 mV s and ν_*se*_ = 4.4 mV s.

In NCSE, besides DRDA, SWDs can often be observed (Ellis, 1978; Granner, 1994; Brenner, 2004; Tay et al., 2006). In the currently used model, SWDs are generated for higher values of cortico-thalamic excitation levels ν_*se*_ as illustrated in Figure 7A. To be more specific, in (Breakspear et al., 2006) the authors demonstrate that SWDs emerge through an inflection point. In Figure 7B the bifurcation diagram from (Breakspear et al., 2006) is replicated. It shows the emergence of delta oscillations through a supercritical Hopf bifurcation for ν_*se*_ ≈ 2 mV s and the emergence of the inflection point for ν_*se*_ ≈ 4.3 mV s leading to 3 Hz SWDs. In (Breakspear et al., 2006), the SWDs were used as a model for generalized absence seizures. In the case of DRDA with intermittent spikes during NCSE, the spikes are the result of thalamo-cortical filtering of afferent stochastic fluctuations, while in the case of SWDs, the spikes are intrinsically generated within the thalamo-cortical system and are integrated into one (pathological) waveform. We thus may interpret the intermittent spikes as a *stochastic precursor* of genuine SWDs. Moreover, this suggests that SWDs during NCSE and generalized absence seizures arise through similar mechanisms.

## Discussion

In this study we have verified the prediction of a computational model of the thalamo-cortical system that the epileptic spikes that can be observed in the EEG of patients during NCSE (Ellis, 1978; Granner, 1994; Uthman and Bearden, 2008) are locked to the phases of the background delta oscillations using EEG data from a single patient. We subsequently used the model to uncover the underlying physiological mechanisms. It is suggested that the spikes originate from inhibitory stochastic fluctuations impinging on thalamo-cortical relay cells, which are transmitted to cortex and observed in the EEG. In particular, although the emergence, morphology, and phase-relationship of the spikes to delta oscillations are shaped by the thalamo-cortical system, it does not generate the spikes intrinsically. The observed phase-locking is a consequence of the fact that relay neurons are excitable only during a narrow temporal window of the delta cycle.

In Phase-locking of epileptic spikes, Physiological mechanism, and Relation to spike-wave discharges we have shown that when the strength of cortico-thalamic excitation is increased, phase-locking becomes stronger and the stochastic spikes deform smoothly into deterministic spikes, which separate the spike and wave of 3 Hz SWDs. In this sense, the spikes observed during NCSE can thus be interpreted as stochastic precursors of SWDs, where the strength of phase-coherence reflects how near the thalamo-cortical system is in generating SWDs. Since these latter are generally interpreted in the context of generalized absence seizures (Robinson et al., 2002; Suffczynski et al., 2004; Breakspear et al., 2006; Rodrigues et al., 2006, 2009; Kim et al., 2009; Marten et al., 2009) we speculate that the SWDs observed during NCSE and generalized absence seizures might share a common mechanism.

The modeling conducted in the present study makes a number of predictions that can be verified experimentally. First, as we have seen in Diffuse rhythmic delta activity it is suggested that the phase-coherence and phase-angle between the spikes and delta oscillations increase and decrease, respectively, as the thalamo-cortical system progresses in the direction of generating SWDs. A direct test of this prediction requires EEG recordings of patients in NCSE that show a gradual transition between DRDA with intermittent spikes and SWDs. Second, in Physiological mechanism we have seen that the epileptic spikes can be observed in cortical pyramidal neurons as well as in neurons within thalamo-cortical relay nuclei. Such a simultaneous involvement of cortex and thalamus has already been demonstrated during generalized SWDs (see (McCormick and Contreras, 2001) for a review). Moreover, they can be observed in thalamo-cortical relay nuclei *before* they become apparent in cortical EEG recordings, something that has already been observed in the WAG/Rij rat model of generalized SWDs (Inoue et al., 1993) and investigated using a detailed biophysical model of the thalamo-cortical system (Destexhe, 1998). Finally, we have suggested that the thalamo-cortical mechanisms underlying NCSE and generalized absence seizures might be similar. If this is true, a third prediction of the model is that the rhythmic delta activity observable during generalized absence seizures, especially in children (Lee and Kirby, 1988) might contain phase-locked spikes. Verification of these predictions allows one to determine to what extent the model captures the key physiological mechanisms involved.

There are a number of possible directions for future research that seem promising. First, following previous modeling studies (Breakspear et al., 2006; Rodrigues et al., 2006, 2009; Marten et al., 2009) epileptiform activity was generated by increasing the cortical-thalamic excitation-level. The physiological basis for this choice is debatable and it cannot be excluded that other parameters also play a role. Thus, a possible future direction of research is a systematic investigation of parameter variations that lead to epileptiform activity. A second possible direction of research is the incorporation of physiological mechanisms that are suspected to underly neuronal damage following prolonged seizure activity (Sankar et al., 1998; Druga et al., 2001). In this way, hypothetical mechanisms derived from recordings in animal models of NCSE can be analyzed quantitatively and allow the formulation of novel predictions. Third, to increase the relevance of the current study to clinical practice, the mechanisms responsable for the relative successfulness of anti-epileptic agents (Claassen et al., 2002) might be investigated by incorporating their pharmacological action into the model, similarly as in Steyn-Ross et al. (2004); Bojak and Liley (2005); Liley and Bojak (2005); Hutt and Longtin (2010); Hindriks and van Putten (2012) in the context of modeling the action of general anaesthetic agents. Such an investigation might point to currently unknown mechanisms by which the thalamo-cortical system can progress into epileptiform activity.

### Conflict of interest statement

The authors declare that the research was conducted in the absence of any commercial or financial relationships that could be construed as a potential conflict of interest.
